# Risk factors for and risk of all-cause and atherosclerotic cardiovascular disease mortality in people with type 2 diabetes and peripheral artery disease: an observational, register-based cohort study

**DOI:** 10.1186/s12933-024-02226-x

**Published:** 2024-04-15

**Authors:** Tarik Avdic, Hanne K. Carlsen, Aidin Rawshani, Soffia Gudbjörnsdottir, Zacharias Mandalenakis, Björn Eliasson

**Affiliations:** 1 Department of Molecular and Clinical Medicine, Institute of Medicine, Sahlgrenska Academy, University of Gothenburg, Medicinaregatan 18G, Gothenburg, 413 45 Sweden; 2 Department of Internal Medicine, Sahlgrenska University Hospital/Östra, Gothenburg, Sweden; 3 Swedish National Diabetes Register, Center of Registers in Region, Gothenburg, Sweden; 4 Department of Medicine, Sahlgrenska University Hospital, Gothenburg, Sweden

**Keywords:** All-cause mortality, Cardiovascular mortality, Peripheral artery disease, Type 2 diabetes mellitus, Cardiovascular risk factors, Cohort study, Observational study

## Abstract

**Background:**

Type 2 diabetes (T2D) and peripheral artery disease (PAD) are recognized as independent risk factors contributing to excess mortality. Contemporary observational studies exploring the associations of risk factors, and risk of all-cause and atherosclerotic cardiovascular disease mortality in persons with T2D following the onset of incident peripheral artery disease are limited. The objectives of this study were to investigate the associations of risk factors, and assess mortality risks in people with T2D compared with controls without T2D after the onset of PAD.

**Methods:**

All persons with T2D (*n* = 150,215) registered in the Swedish National Diabetes Register between 2005 and 2009 were included, along with 346,423 controls without T2D matched for sex and age. Data were retrieved from several national registries, capturing information on risk factors, onset of incident peripheral artery disease, other comorbidities, socioeconomic factors, and outcomes. To compare persons with T2D and controls following the onset of peripheral artery disease regarding the risk of all-cause, and atherosclerotic cardiovascular disease mortality, Cox proportional hazard models and Kaplan-Meier curves were employed. A gradient-boosting model was utilized to estimate the relative statistical contribution of risk factors to the modeling of incident mortality risk in people with both T2D and peripheral artery disease.

**Results:**

Crude rates of incident all-cause mortality were higher in individuals with T2D compared with controls, following the onset of PAD (600.4 (95% CI, 581.4-619.8) per 10,000 person-years versus 549.1 (95% CI, 532.1-566.5) per 10,000 person-years). Persons with T2D had an adjusted hazard ratio (HR) for all-cause mortality of 1.12 (95% CI, 1.05–1.19, *P* < 0.01) compared with controls after onset of incident PAD. The comparable adjusted HR for cardiovascular mortality was 1.13 (95% CI, 1.07–1.19, *P* < 0.01). High age and hyperglycemia at baseline played a significant role in contributing to the predictive models for incident all-cause and cardiovascular mortality among individuals with both T2D and PAD.

**Conclusions:**

The presence of T2D with concomitant PAD is related to an increased risk of both all-cause and cardiovascular mortality compared with individuals with only PAD. This argues for implementing optimized and intensive treatment strategies for individuals with both conditions.

**Supplementary Information:**

The online version contains supplementary material available at 10.1186/s12933-024-02226-x.

## Background

Peripheral artery disease (PAD) serves as an indicative marker of widespread atherosclerosis, and individuals with PAD face a significantly increased risk of experiencing other atherosclerotic cardiovascular diseases (ASCVD), as well as increased susceptibility to ASCVD and all-cause mortality. (1–2) The prevalence of PAD is on an upward trajectory, attributed to an aging population living longer with chronic illnesses and prolonged exposure to well-established risk factors for PAD, including smoking and type 2 diabetes Mellitus (T2D). (3–4) Findings from a recent British cohort study underscore PAD as one of the most prevalent initial vascular complications in persons with T2D [5]. Furthermore, the risk of PAD is 2 to 4 times higher in people with T2D than in the general population, (6–7) and PAD manifests more severely in persons with T2D compared with the general population [6]. 

Despite epidemiological analyses indicating a decline in the risk and rates of ASCVD and mortality among persons with T2D compared with the general population, (8–9) T2D is still considered a risk factor for excess mortality. (10–11) The observed positive trends in ASCVD risk and mortality rates among individuals with T2D are likely the result of improved coordinated care, emphasizing lifestyle modifications and enhanced management of hyperglycemia, blood pressure, and dyslipidemia [12–14]. However, it remains less well-established whether similar trends in risk and rate reduction of ASCVD and all-cause mortality extend to individuals managing both T2D and PAD.

Previous analyses examining the risk of mortality in persons with both T2D and PAD have indicated a contrasting trend. These findings suggest that individuals with both T2D and PAD face a higher risk of mortality compared to those with only PAD. These prior analyses may have limitations such as not being contemporary, not distinguishing between type 1 diabetes mellitus (T1D) and T2D, and having small sample sizes. (15–16)

The overarching goal of the present study was to build upon and provide updated insights compared to previous reports, focusing on a contemporary cohort of individuals with T2D. The primary aim of the study was to assess the additional extent to which T2D is associated with the risk of incident all-cause and ASCVD mortality after the onset of incident PAD. This included exploratory comparisons of mortality risks between individuals with T2D and controls, where the latter were matched for sex, age, and county but not for risk factor status. The secondary aim involved investigating the relative importance of various risk factors in modeling the prediction of incident all-cause and ASCVD mortality risk among individuals with T2D and PAD.

## Methods

This observational study is conducted within a register-based framework, utilizing data from several Swedish national health and administrative registers. The linking of data is facilitated through the personal identification number, a unique identifier assigned to each Swedish citizen. The registers incorporated in this study comprise the Swedish National Diabetes Register (NDR) [17], the Total Population Register (TPR) (administered by the government agency Statistics Sweden) [18], the National Patient Register (NPR) [19], the Prescribed Drug Register (PDR) [20], the Swedish Cause of Death Register [21], and the Longitudinal Integration Database for Health Insurance and Labor Market Studies (LISA) [22]. 

### Study cohort

The NDR has been thoroughly documented in previous literature [4, 10, 17]. In summary, established in 1996, the NDR boasts nationwide coverage and includes nearly all individuals with T2D in Sweden. On an annual basis, data encompassing patient characteristics, risk factors, treatment, and complications related to diabetes mellitus are collected from patient encounters at healthcare facilities specializing in the management of people with T2D, and reported to the NDR.

The epidemiological definition of T2D (dietary treatment with or without the use of oral antihyperglycemic medication; and additionally, individuals diagnosed with diabetes mellitus at the age of 40 years or older and treated with insulin, either exclusively or in combination with other oral antihyperglycemic agents, are also categorized as having T2D) was used to identify persons with T2D from the NDR in this study.

The study cohort consisted of all persons with T2D who had at least one registration in the NDR between January 1, 2005, and December 31, 2008. At the initial registration in the NDR, which also served as the index date for the study, each person with T2D was matched for sex, age, and county with two control participants without T2D. The controls were randomly sampled with replacement from the general population (Total Population Register, Statistics Sweden), and the primary objective of the matching process was to secure controls without T2D for use as matched comparators. The control participants were initially free of T2D at the time of their selection, but a very small subset of these individuals developed T2D during the wash-in period (2005–2008) before follow-up. Instead of censoring them, these cases were matched with two new controls, resulting in a non-integer case-control ratio. Controls who received a T2D diagnosis in the NPR during follow-up were censored at the time of diagnosis. Study participants with manifest PAD or amputation at baseline were excluded from the present analyses.

The subset of individuals with T2D and controls who experienced onset of incident PAD diagnosis during the study period had their follow-up initiated at the time of the registered incident PAD diagnosis in the NPR. Their baseline data were then set as the last pre-PAD observation, encompassing sex, age, and all relevant comorbidities and socioeconomic factors listed below. Study participants were followed until death, covering both all-cause mortality and ASCVD mortality, or until the conclusion of the study period on December 31, 2020.

### Baseline data and PAD exposure during the study period

Baseline comorbidity data and the identification of incident PAD during the study period were acquired from the NPR, which employs Swedish revisions of *International Classification of Diagnoses* (ICD) version 10 codes, for diagnosing and classifying medical conditions. Administered by the Swedish National Board of Health and Welfare, the NPR serves as the source for this health-related data [19]. A detailed description and definition of all comorbidities, along with their corresponding ICD-10 codes considered in this study, are provided in Supplementary Table 1, available in the online data supplement. The definition of ASCVD in this study encompasses a combination of myocardial infarction, ischemic heart disease, and stroke. Socioeconomic factors such as marital status, educational level, region of origin, and income level were gathered from the LISA, administered by Statistics Sweden [22]. The PDR facilitated the collection of data on filled prescriptions for statins, antihypertensive, antiplatelet, and anticoagulant medications over a one-year period preceding the index date [20]. The methodology of defining PAD, other comorbidities and outcomes using corresponding ICD-10 codes in the NPR, along with the collection of additional baseline and outcome data from various Swedish national health and administrative registers, has been employed in prior national and international collaborations [4, 8, 10, 23, 24]. 

### Risk factors for mortality among individuals with T2D and PAD

Clinical characteristics and cardiovascular risk factors for persons with T2D were gathered from the NDR. In the analyses exploring the associations of risk factors with all-cause and ASCVD mortality in individuals with T2D after the onset incident PAD, the following baseline variables were considered: glycated haemoglobin (HbA1c), blood pressure levels (systolic and diastolic), total cholesterol, low-density lipoprotein cholesterol (LDL-C), high-density lipoprotein cholesterol (HDL-C), triglycerides, body mass index (BMI), smoking status (defined as a current smoker at study entry), presence of micro- and macroalbuminuria, and estimated glomerular filtration rate (eGFR). The eGFR was calculated using the MDRD equation [25]. Microalbuminuria was defined as two separate samples of urine albumin/creatinine ratio of 3–30 mg/mmol (30–300 mg/g) within a year, while macroalbuminuria followed a similar definition but with higher ratios than 30 mg/mmol. Risk factor data were not available for control subjects in this study.

### Outcomes

The two pre-defined main outcomes in this study were incident all-cause mortality and ASCVD mortality (defined with ICD-codes I00-I99, R570, R960, R961). Information on these outcomes were obtained from the Swedish Cause of Death Register, administered by the Swedish National Board of Health and Welfare. This register contains comprehensive details on all deaths, including causes and timing of death, for individuals registered in Sweden. The underlying cause of death is coded based on ICD codes, and reporting to this register is mandatory [21]. Both people with T2D and controls had their outcomes equally ascertained.

### Statistical analyses

Descriptive statistics are reported using means with standard deviations (SD) or, when appropriate, medians with interquartile range (IQR) values for continuous variables. Categorical variables are presented as counts (n) with corresponding percentages (%) for categorical variables. The magnitude of differences at baseline across variables between study groups, were calculated with standardized mean differences (SMD), where values below 0.1 were considered non-significant.

Event rates were calculated as the number of incident all-cause mortality and ASCVD mortality events per person-years for individuals with T2D and controls. Event rates in individuals without PAD are reported per PAD-free year. At time of PAD-diagnosis, the individuals are censored. For individuals with PAD, event rates are reported per PAD-exposed year. Stratification was performed based on whether onset of incident PAD occurred during the study period or not, and sex aswell. These rates were presented with 95% exact Poisson confidence intervals (CI) and visualized using Kaplan-Meier and cumulative incidence curves.

The risk of incident all-cause mortality and ASCVD mortality was further assessed by employing Cox proportional hazards regression. A competing risk approach was applied for ASCVD mortality using the Fine-Gray method. These models were also stratified on whether onset of incident PAD diagnosis occurred or not in a multi-state design. The comparison of risk was conducted between individuals with T2D and controls. Specifically, the risk comparison was divided into two sub analyses: one sub analyses where the risk was compared between individuals with T2D and controls who had experienced onset of incident PAD diagnosis during the study period, and the other sub analyses where the risk was compared between those individuals with T2D and controls who did not experience the onset of incident PAD. This approach allowed for a more nuanced evaluation of risk differences in these distinct subgroups. The Cox proportional hazards models underwent a stepwise adjustment process. The final model encompassed adjustments for age, sex, income level, educational level, marital status, region of origin, selected medications (antihypertensive medication, statins, anticoagulant medication, antiplatelet therapy), and comorbidities as defined in Supplementary Table 1. The associations are summarized as hazard ratios (HR) presented with 95% confidence intervals (CIs).

The secondary objective, focused on evaluating T2D-related ASCVD risk factors and their associations with all-cause and ASCVD mortality among persons with T2D after the onset of incident PAD, was analysed using Cox proportional hazards testing. To assess the relative importance of T2D-related cardiovascular risk factors in the models estimating the risk of all-cause and ASCVD mortality, a Gradient-Boosting Model (GBM) with a proportional hazards loss function was employed [26]. The GBM-model was fitted with a shrinkage of 0.05 for computational efficiency and an interaction depth of 3. The number of trees, optimized at 1167, was determined through 5-fold cross-validation. The relative importance, also known as the partial graded effect, of each predictor to the model for the outcome risk is presented as an estimate. This estimate, termed relative importance, indicates the predictive significance of each risk factor to the model for outcomes risk and was determined using GBM, a machine learning technique.

Given the register-based nature of this study, missing data on T2D-related cardiovascular risk factors in the NDR were acknowledged. Presumed to be missing at random, a multiple imputation model—multiple imputations by chained equations (MICE)—was applied to handle missing data, mainly clinical biomarkers from the NDR. This iterative imputation method predicted missing values using observed data, and the imputed datasets were combined to create a final dataset for subsequent analyses. The imputed data was used the assessment of mortality risk as well as the evaluation of risk factor importance to the models and association of risk factors with outcomes. Supplementary Table 2 provides a comprehensive list of variables considered during the imputation process, ensuring transparency in the imputation model.

The assessment of Schoenfeld’s and Martingale residuals indicated that the proportional hazard assumption was met. All statistical testing adhered to a two-sided significance level of 5%, and 95% confidence intervals (CIs) were employed. Given the exploratory nature of the study, no correction for multiple comparisons was applied. The statistical analyses were conducted using R version 4.0.3.

## Results

### Baseline characteristics of study participants

The study cohort comprised 150,215 persons with T2D and 346,423 matched controls without T2D, with baseline characteristics presented in Table 1 for the overall study cohort, as well as stratified by incident PAD during study period. At baseline, the mean age was slightly higher among individuals with T2D compared with controls (65.7 versus 64.1 years). The T2D cohort exhibited nearly twice the prevalence of co-existing ASCVD comorbidities compared with controls, and were more frequently treated with antihypertensive medications, statins, antiplatelet, and anticoagulant medications.

**Table 1:** Baseline characteristics for individuals with type 2 diabetes and controls

Parameter	All Controls (*n* = 346 423)	All T2D (*n* = 150 215)	Controls, no PAD (*n* = 338 976)	T2D, No PAD (*n* = 143 578)	Controls with PAD (7 447)	T2D with PAD (6 637)	SMD
Age, y	64.05 (14.17)	65.69 (12.09)	63.90 (14.23)	65.52 (12.18)	70.58 (8.92)	69.23 (9.46)	0.13
Age category, n (%)							
18–44 years	30 654 (8.8)	7 318 (4.9)	30 638 (9.0)	7 279 (5.1)	16 (0.2)	39 (0.6)	
45–54 years	43 080 (12.4)	18 649 (12.4)	42 815 (12.6)	18 290 (12.7)	265 (3.6)	359 (5.4)	
55–64 years	93 362 (27.0)	42 592 (28.4)	91 704 (27.1)	40 870 (28.5)	1 658 (22.3)	1 722 (25.9)	
65–74 years	95 044 (27.4)	43 549 (29.0)	92 129 (27.2)	41 083 (28.6)	2 915 (39.1)	2 466 (37.2)	
≥ 75 years	84 283 (24.3)	38 107 (25.4)	81 690 (24.1)	36 056 (25.1)	2 593 (34.8)	2 051 (30.9)	
Women, n (%)	153 050 (44.2)	66 623 (44.4)	149 951 (44.2)	64 010 (44.6)	3 099 (41.6)	2 613 (39.4)	0.003
Marital status, n (%)							0.06
Single	56 948 (16.4)	24 680 (16.4)	55 469 (16.4)	23 438 (16.3)	1 479 (19.9)	1 242 (18.7)	
Married	184 446 (53.2)	81 521 (54.3)	180 534 (53.3)	77 983 (54.3)	3 912 (52.5)	3 538 (53.3)	
Divorced	57 390 (16.6)	22 006 (14.7)	56 671 (16.7)	21 287 (14.8)	719 (9.7)	719 (10.8)	
Widowed	47 626 (13.7)	22 004 (14.6)	46 291 (13.7)	20 866 (14.5)	1 335 (17.9)	1 138 (17.1)	
Educational level, n (%)							0.2
Compulsory school	125 470 (36.8)	64 481 (43.8)	121 994 (36.6)	61 222 (43.5)	3 476 (47.3)	3 259 (50.0)	
Upper secondary school	137 939 (40.4)	59 811 (40.7)	135 097 (40.5)	57 318 (40.8)	2 842 (38.7)	2 493 (38.2)	
College/university	77 677 (22.8)	22 829 (15.5)	76 648 (23.0)	22 060 (15.7)	1 029 (14.0)	769 (11.8)	
Income quartile, n (%)							0.19
1st quartile	81 365 (23.8)	40 693 (27.3)	79 389 (23.8)	38 822 (27.3)	1 976 (26.7)	1 871 (28.3)	
2nd quartile	79 829 (23.4)	41 236 (27.7)	77 479 (23.2)	39 109 (27.5)	2 350 (31.7)	2 127 (32.1)	
3rd quartile	85 965 (25.2)	36 987 (24.8)	84 166 (25.2)	35 425 (24.9)	1 799 (24.3)	1 562 (23.6)	
4th quartile	94 103 (27.6)	29 977 (20.1)	92 815 (27.8)	28 916 (20.3)	1 288 (17.4)	1 061 (16.0)	
Country/Region of birth, n (%)							0.15
Sweden	301 745 (87.1)	123 267 (82.1)	295 242 (87.1)	117 725 (82.0)	6 503 (87.3)	5 542 (83.5)	
Europe	16 393 (4.7)	8 624 (5.7)	15 950 (4.7)	8 152 (5.7)	443 (5.9)	472 (7.1)	
Rest of the world	28 285 (8.2)	18 324 (12.2)	27 784 (8.2)	17 701 (12.3)	501 (6.7)	623 (9.4)	
History of comorbidities, n (%)							
ASCVD	35 758 (10.3)	27 863 (18.5)	33 984 (10.0)	25 765 (17.9)	1 774 (23.8)	2 098 (31.6)	0.19
Ischemic heart disease	30 560 (8.8)	22 179 (14.8)	29 190 (8.6)	20 573 (14.3)	1 370 (18.4)	1 606 (24.2)	0.24
Myocardial Infarction	16 793 (4.8)	13 794 (9.2)	15 917 (4.7)	12 717 (8.9)	876 (11.8)	1 077 (16.2)	0.17
Stroke	15 981 (4.6)	9 972 (6.6)	15 366 (4.5)	9 291 (6.5)	615 (8.3)	681 (10.3)	0.09
Atrial fibrillation	19 822 (5.7)	12 669 (8.4)	19 012 (5.6)	11 884 (8.3)	810 (10.9)	785 (11.8)	0.11
Heart failure	12 996 (3.8)	10 384 (6.9)	12 434 (3.7)	9 700 (6.8)	562 (7.5)	684 (10.3)	0.14
Valvular disease	6 071 (1.8)	3 255 (2.2)	5 848 (1.7)	3 031 (2.1)	223 (3.0)	224 (3.4)	0.03
Acute kidney failure and Chronic kidney disease	2 708 (0.8)	1 815 (1.2)	2 542 (0.7)	1 647 (1.1)	166 (2.2)	168 (2.5)	0.04
Psychiatric disease	11 877 (3.4)	6 615 (4.4)	11 658 (3.4)	6 417 (4.5)	219 (2.9)	198 (3.0)	0.05
Cancer	35 483 (10.2)	16 183 (10.8)	34 565 (10.2)	15 438 (10.8)	918 (12.3)	745 (11.2)	0.02
Age at T2D diagnosis, y	-	60.28 (12.13)	-	60.20 (12.18)	-	61.98 (10.90)	
T2D duration, y	-	4.87 (6.20)	-	4.80 (6.14)	-	6.58 (7.11)	
HbA1c, mmol/mol	-	52.64 (13.60)	-	52.54 (13.57)	-	54.91 (13.99)	
Blood pressure, mm Hg							
Systolic	-	138.95 (17.56)	-	138.77 (17.49)	-	142.88 (18.54)	
Diastolic	-	78.10 (9.83)	-	78.17 (9.82)	-	76.72 (9.88)	
LDL-C, mmol/L	-	2.87 (0.94)	-	2.87 (0.93)	-	2.84 (0.98)	
HDL-C, mmol/L	-	1.30 (0.41)	-	1.30 (0.41)	-	1.27 (0.40)	
Total cholesterol, mmol/L	-	4.98 (1.08)	-	4.98 (1.08)	-	4.99 (1.11)	
Triglycerides, mmol/L	-	1.89 (1.28)	-	1.88 (1.28)	-	2.03 (1.30)	
BMI, kg/m^2^	-	29.75 (5.32)	-	29.80 (5.34)	-	28.80 (4.66)	
eGFR, mL·min^− 1^/1.73 m^− 2^	-	80.66 (23.34)	-	80.84 (23.30)	-	76.69 (23.82)	
Albuminuria, n (%)							
Microalbuminuria	-	14 928 (15.4)	-	14 060 (15.2)	-	868 (20.1)	
Macroalbuminuria	-	378 (0.4)	-	364 (0.4)	-	14 (0.3)	
Smoker, n (%)	-	19 130 (15.3)	-	17 636 (14.7)	-	1 494 (26.7)	
Medication, n (%)							
Antihypertensive	126 831 (36.6)	96 516 (64.3)	122 451 (36.1)	91 650 (63.8)	4 380 (58.8)	4 866 (73.3)	0.58
Antiplatelet	67 799 (19.6)	53 499 (35.6)	64 972 (19.2)	50 277 (35.0)	2 827 (38.0)	3 222 (48.5)	0.37
Statins	54 158 (15.6)	60 696 (40.4)	51 767 (15.3)	57 421 (40.0)	2 391 (32.1)	3 275 (49.3)	0.57
Anticoagulant medication	13 092 (3.8)	8 524 (5.7)	12 554 (3.7)	7 964 (5.5)	538 (7.2)	560 (8.4)	0.09
Antihyperglycemic therapy, n (%)							
Dietary	-	55 843 (37.2)	-	53 957 (37.6)	-	1886 (28.4)	
Oral agents	-	65 588 (43.7)	-	62 644 (43.6)	-	2 944 (44.4)	
Insulin	-	13 159 (8.8)	-	12 337 (8.6)	-	822 (12.4)	
Insulin and oral agents	-	15 625 (10.4)	-	14 640 (10.2)	-	985 (14.8)	

Baseline characteristics for all persons with type 2 diabetes and all controls, as well as stratified whether onset of incident peripheral artery disease occurred or not. Data are presented as mean (standard deviation) or n (%). ASCVD indicates atherosclerotic cardiovascular disease; BMI, body mass index; eGFR, estimated glomerular filtration rate; HbA1c, glycated hemoglobin; HDL-C, high-density lipoprotein cholesterol; LDL-C, low-density lipoprotein cholesterol; MI, myocardial infarction; PAD, Peripheral artery disease; SMD, Standardized mean difference; and T2D, type 2 diabetes mellitus

During the study period, 6,637 individuals with T2D (4.4%) and 7,447 controls (2.2%) developed incident PAD (Table 2). Those with T2D and PAD were older at baseline and had a higher prevalence of coexisting ASCVD comorbidities compared with those with T2D without PAD. Similar trends, albeit more accentuated, were observed in controls when stratified for PAD.

**Table 2:** All-cause and atherosclerotic cardiovascular mortality in individuals with type 2 diabetes versus controls

Group	No. of Events	Person-Years	Incidence Rate per 10 000 Person-Years (95% CI)	Hazard Ratio (95% CI)*	*P*-Value *P* < 0.05 = †
**All-cause mortality**					
Controls, no PAD	116 414	3 681 164	316.2 (314.4–318.1)	Reference	
T2D, no PAD	60 558	1 539 673	393.3 (390.2–396.5)	1.11 (1.09–1.13)†	*P* < 0.05
Controls with PAD	3 935	71 661	549.1 (532.1–566.5)	Reference	
T2D with PAD	3 804	63 361	600.4 (581.4–619.8)	1.12 (1.05–1.19)†	*P* < 0.05
**Cardiovascular mortality**					
Controls, no PAD	69 637	3 681 164	189.2 (187.8–190.6)	Reference	
T2D, no PAD	39 885	1 539 673	259.1 (256.5–261.6)	1.09 (1.08–1.10)†	*P* < 0.05
Controls with PAD	2 971	71 661	414.6 (399.8–429.8)	Reference	
T2D with PAD	2 959	63 361	467 (450.3–484.1)	1.13 (1.07–1.19)†	*P* < 0.05

Number of events, incidence rates and adjusted hazard ratios for mortality (all-cause and atherosclerotic cardiovascular disease mortality) in individuals with type 2 diabetes versus controls, stratified by whether onset of incident peripheral artery disease occurred or not

*Based on Cox regression analysis adjusted for age, sex, diabetes duration, all demographic and socioeconomic variables, selected medications and comorbidities shown in Table 1

†Significant difference versus controls (*P* < 0.05)

CI indicates confidence interval; PAD, peripheral artery disease; and T2D, type 2 diabetes mellitus

### All-cause mortality

Table 2 summarizes incident all-cause and cardiovascular mortality events during follow-up, provides crude mortality rates with 95% CIs, and adjusted HRs for all-cause and ASCVD mortality. Over the follow-up period, there were 3,804 all-cause mortality events among individuals with T2D who experienced incident PAD during the study period, 3,935 events among controls experiencing PAD, 60,558 events among individuals with T2D without PAD, and 116, 414 events in controls without PAD.

Crude incidence rates of all-cause mortality were highest for individuals with T2D experiencing PAD, at 600.4 (95% CI, 581.4-619.8) per 10 000 person-years, followed by controls experiencing PAD (549.1, 95% CI, 532.1-566.5), individuals with T2D without PAD (393.3, 95% CI, 390.2-396.5), and controls without PAD (316.2, 95% CI, 314.4-318.1) per 10 000 person-years. The Kaplan-Meier survival curves highlight that PAD, irrespective of T2D, is a significant marker for all-cause mortality. Roughly 40–50% of those persons with T2D as well as controls who experienced onset of incident PAD during the study period, were deceased within 10 years of follow-up. The corresponding figures were 20–30%. for those persons with T2D and controls, without concomitant PAD. Similar tendencies are observed when the cohort is stratified for sex. For all Kaplan-Meier curves, please refer to supplementary Figs. 1–6.

Among those individuals with T2D and controls who experienced incident PAD during study period, the adjusted HR for all-cause mortality was 1.12 (95% CI, 1.05–1.19, *P* < 0.01) in persons with T2D compared with controls. The comparable adjusted HR for all-cause mortality was 1.11 (95% CI, 1.09–1.13, *P* < 0.01) when PAD was not present (Table 2).

### ASCVD mortality

Similar to the trends observed for all-cause mortality, the study found similar patterns for ASCVD mortality. During the study period, there were 2,959 ASCVD mortality events among individuals with T2D experiencing incident PAD, 2,971 events among controls experiencing PAD, 39,885 events among persons with T2D without PAD, and 69,637 events in controls without PAD. (Table 2)

The crude incidence rates of ASCVD mortality were highest for individuals with T2D developing incident PAD at 467 (95% CI, 450.3-414.1) per 10,000 person-years, followed by controls developing incident PAD (414.6, 95% CI, 399.8-429.8), individuals with T2D without PAD, and controls without PAD. Approximately 35–40% of individuals with T2D and controls developing incident PAD during the study period experienced ASCVD mortality within 10 years of follow-up, as illustrated by the Kaplan-Meier curves. The corresponding figures when PAD was not present were 15–20%. (Supplementary Fig. 2)

The adjusted excess risk of ASCVD mortality in individuals with T2D compared with controls was HR 1.13 (95% CI, 1.07–1.19, *P* < 0.01), when incident PAD was present in both groups. Devoid of PAD presence, the comparable adjusted excess risk in persons with T2D compared with controls was HR 1.09 (95% CI, 1.08–1.10, *P* < 0.01).

### Risk factors for all-cause – and cardiovascular mortality in those with T2D and PAD

Risk factors associated with a higher risk of incident all-cause mortality and ASCVD mortality among persons with T2D after onset of incident PAD are outlined in supplementary Tables 3 and supplementary Table 4, with all of the adjusted HRs obtained from Cox analyses. Risk factors associated with higher incident all-cause mortality included coexisting conditions such as acute kidney failure and chronic kidney disease (HR 1.55, 95% CI, 1.27–1.9; *P* < 0.01) and heart failure (HR 1.5, 95% CI 1.34–1.67; *P* < 0.01). Treatment with antihypertensive medication (HR 1.29, 95% CI 1.17–1.42; *P* < 0.01), insulin treatment (HR 1.26, 95% CI 1.1–1.43; *P* < 0.01), and smoking (HR 1.32, 95% CI 1.21–1.44; *P* < 0.01) were also identified as risk factors associated with a higher risk of incident all-cause mortality among persons with T2D and incident PAD. (Supplementary Table 3) Risk factor associations and adjusted HR magnitudes for ASCVD mortality within the same cohort were found to be near identical to those for all-cause mortality. (Supplementary Table 4)

**Fig. 1:**
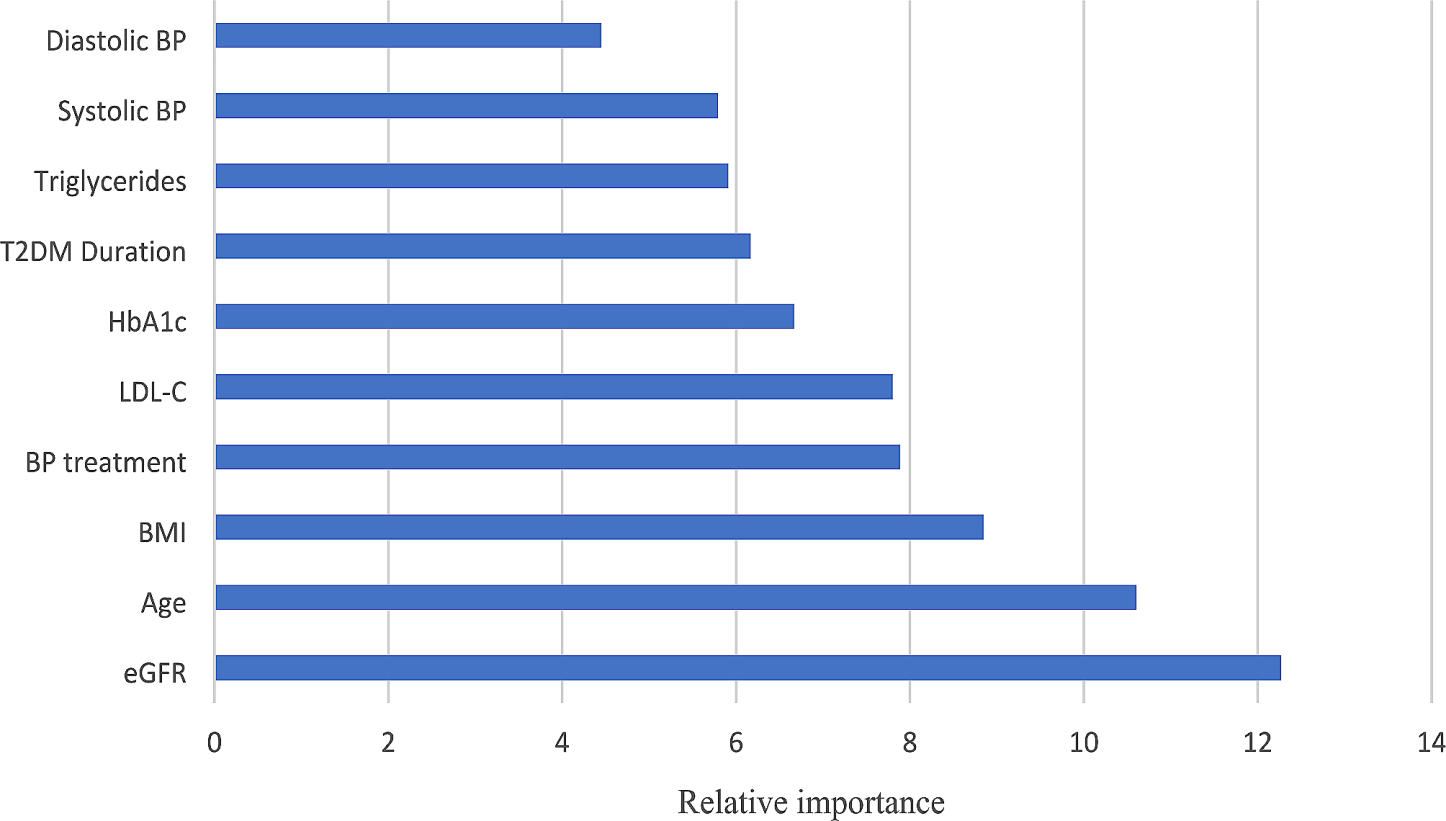
Predictors/Relative importance of different risk factors for all-cause mortality in people with type 2 diabetes and peripheral artery disease The relative importance of different risk factors to the multivariable gradient boosting model for incident all-cause mortality after onset of incident peripheral artery disease in people with type 2 diabetes. BMI indicates body mass index; T2DM Duration: type 2 diabetes duration; BP: blood pressure; eGFR: estimated glomerular filtration rate; HbA1c: glycated hemoglobin; LDL-C: low-density lipoprotein cholesterol

**Fig. 2:**
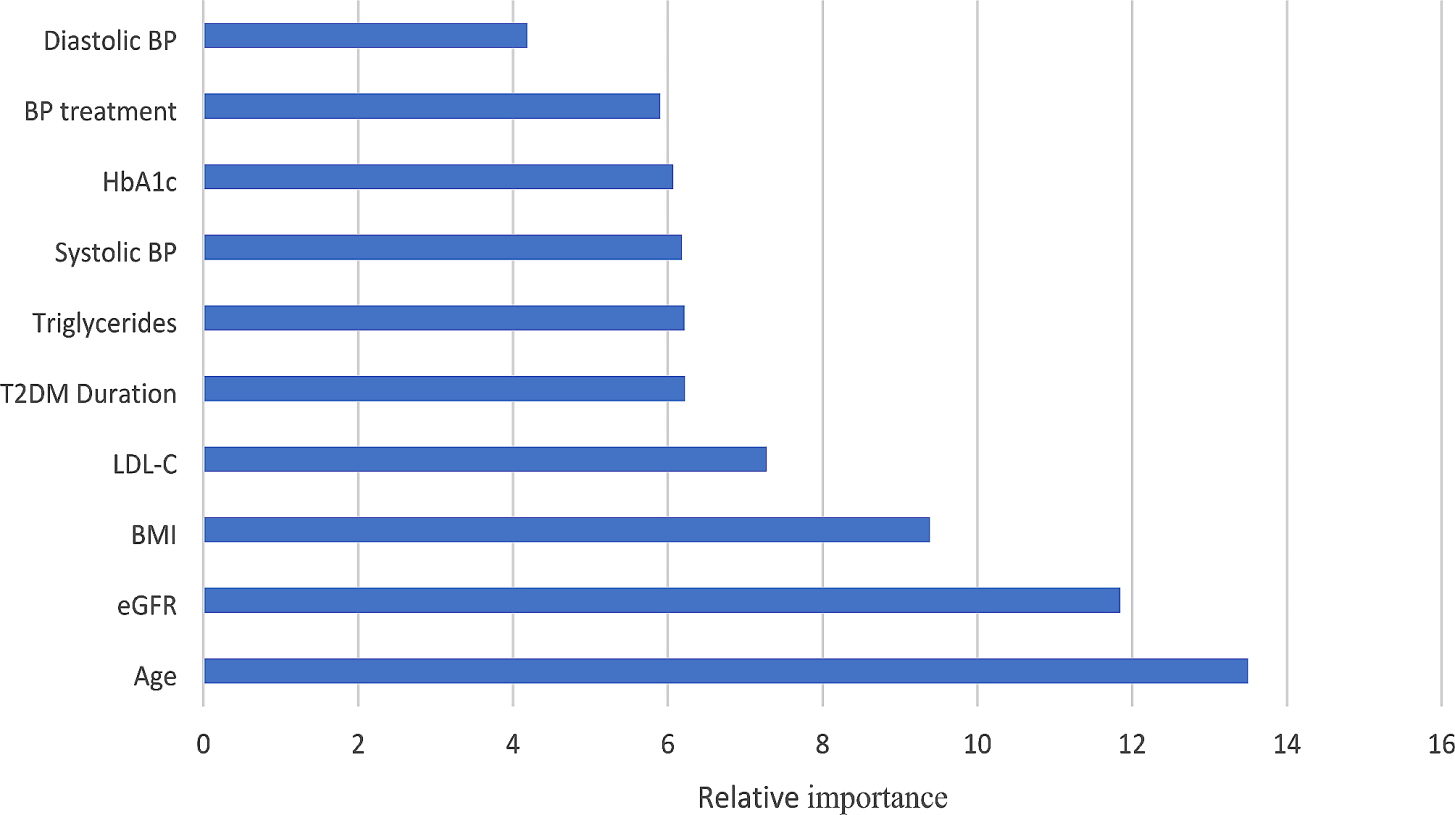
Predictors/ relative importance of different risk factors for atherosclerotic cardiovascular disease mortality in people with type 2 diabetes and peripheral artery disease The relative importance of different risk factors to the multivariable gradient boosting model for incident atherosclerotic cardiovascular disease mortality after onset of incident peripheral artery disease in people with type 2 diabetes. BMI indicates body mass index; T2DM Duration: type 2 diabetes duration; BP: blood pressure; eGFR: estimated glomerular filtration rate; HbA1c: glycated hemoglobin; LDL-C: low-density lipoprotein cholesterol

Figures 1 and 2 illustrate the relative variable importance of various risk factors in predicting incident all-cause mortality and ASCVD mortality among individuals with T2D and concomitant PAD, derived from the GBM-model. The risk factors with the greatest relative importance for all-cause mortality were income, eGFR, age, BMI, antihypertensive medication, LDL-C, HbA1c, T2D duration, triglycerides, systolic blood pressure, and diastolic blood pressure (descending order) (Fig. 1). For ASCVD mortality, age, eGFR, BMI, LDL-C, T2D duration, triglycerides, systolic blood pressure, HbA1c, antihypertensive medication, and diastolic blood pressure (in descending order) were considered the strongest predictors. (Fig. 2)

## Discussion

This observational study illustrates the role of PAD as a substantial predictor of all-cause and ASCVD mortality, regardless of T2D status. Unsurprisingly, persons with T2D and concomitant incident PAD exhibited the highest crude rates for both outcomes, with rates in controls without T2D but with PAD nearly paralleling those in the T2D and PAD group. In the subset of study participants developing incident PAD, individuals with T2D, faced an adjusted 12% excess relative risk for all-cause mortality and a 13% excess relative risk for ASCVD mortality compared with controls. Age, hyperglycemia, traditional cardiovascular risk factors (hypertension, dyslipidemia, high BMI, smoking), impaired kidney function, and baseline ASCVD comorbidity emerged as significant contributors to predictive models for mortality (both all-cause and ASCVD mortality) in individuals with T2D and PAD. These insights underscore that people with T2D and concomitant PAD have high mortality risks and suggest the need of early and intensive management of ASCVD risk factors in these persons.

The data suggesting independent associations of T2D and PAD with heightened risks of all-cause and ASCVD mortality, aligns with existing substantial evidence [1, 2, 6, 8–10]. Despite the well-established association between T2D and PAD [3–8], there has been limited exploration into mortality risks in individuals managing both diabetes mellitus (DM) and PAD [15, 16, 27, 28]. Earlier studies have hinted at a greater risk of all-cause mortality in those with DM and PAD compared to individuals with PAD alone [15, 16, 27]. The current analyses meaningfully extend these previous findings, and shed light on the potential significance of early and comprehensive ASCVD risk factor management to mitigate the risk of mortality (both all-cause and ASCVD) in those with T2D and concurrent PAD. While the general trend in crude mortality rates and adjusted HRs aligns with prior findings, the specific estimates in this study differ compared with those from other studies [15, 16, 27]. The observed variations in point estimates between this study and previous research can be ascribed to several factors. Firstly, discrepancies arise from differences in the studied populations, variations in the time frames of the investigations, and distinctions in the definitions of PAD and DM. Earlier studies often failed to differentiate between type 1 diabetes mellitus and T2D [15, 16, 27]. Secondly, recent evidence suggests a decline in the risk and rates of ASCVD, ASCVD mortality, and all-cause mortality in individuals with T2D [8, 9, 29]. This decline may be attributed to improved organized care, with intensive multifactorial ASCVD risk factor control, enhanced lifestyle interventions, and pharmacological treatments [12–14, 30–32]. These advancements in diabetes care are most likely reflected in the point estimates of the present study.

The observed 12% excess relative risk for all-cause mortality and 13% excess relative risk for ASCVD mortality in persons with T2D compared with controls, within the subset of study participants developing incident PAD during follow-up, underscores the likely association between hyperglycemia and mortality risk (both all-cause and ASCVD), as has been suggested by prior studies [15, 16, 27, 28]. Hyperglycemia and long diabetes duration may lead to an increased glycemic load and vascular damage within several vascular beds [33], resulting in a combination of a more severe PAD progression [27] and a widespread vascular damage in several other arteries, contributing to increased mortality risks (both all-cause and ASCVD mortality). Additionally, various other mechanisms associated with T2D, challenging or uncommon to measure in observational studies, may have also contributed to increased risks, including insulin resistance, inflammation, excess central adiposity, and hypercoagulability, among others. (34–35) Oxidative stress and inflammation are prevalent characteristics observed in both T2D and PAD individually. While these factors were not directly measured in the present study, their presence may have contributed to the high observed crude rates of all-cause and ASCVD mortality among study participants with PAD alone, that suggested PAD as a significant marker for mortality risk [36]. The observed high mortality risks, encompassing both all-cause and ASCVD-related mortality, in individuals managing both T2D and PAD, underscore the critical need for early detection and intensive management of ASCVD risk factors in these patients within healthcare settings.

Present analyses examining the association between risk factors and mortality risks (both all-cause and ASCVD), in persons with T2D and PAD, suggest age, hyperglycemia, traditional ASCVD risk factors (such as hypertension, smoking, dyslipidemia), and coexisting ASCVD comorbidity at baseline, to be important factors associated with outcomes. Limited prior data exists on the association between these risk factors and mortality outcomes in this patient population, making comparisons challenging. A smaller community-based study is the only available equivalent [27], and the present results align with those from this previous study. The findings that there is a strong association between impaired kidney function and mortality in persons with type 2 diabetes and PAD is debated, but may to some extent be explained by contrast-induced nephropathy in those undergoing surgical PAD interventions [37]. Comparing these findings with prior results on associations between all-cause mortality risk and ASCVD risk factors in individuals with solely T2D, it appears that the risk factor profile varies [9]. 

### Strengths and limitation

This observational study has several strengths. It benefits from a large and unselected sample of individuals with T2D, minimizing the risk of selection bias. The study is contemporary, offering insights into the current consequences of advanced diabetes complications in clinical practice. The data is sourced from high-quality nationwide health registers with extensive coverage [19, 38]. However, there are notable limitations. Risk factor data were unavailable for the control group. The impact of the non-integer case-control due to very few controls developing T2D during the wash-in period did not affect the result in any meaningful way. Analyses examining risk factor associations with mortality risks were based on imputed baseline data; and some may consider it a limitation that baseline data is not be representative of follow-up. Nonetheless, utilizing imputed baseline covariates, given data is missing at random, is often preferred from a clinical perspective, as it reflects the initial status of risk factors at the start of the study. The register-based approach relies on ICD codes, lacking details on symptoms, extent, and severity of PAD events, potentially affecting accuracy of PAD diagnosis. Residual confounding is inherently plausible in observational studies, and study results may vary dependent on preferred choice of statistical methods. Importantly, the study is observational, and analyses of risk factor associations are limited to assessing the statistical strength of associations. Clinical implications on management of risk factors were not evaluated with present study design.

## Conclusions

Present analyses demonstrated increased risk of all-cause mortality and ASCVD mortality in persons with T2D compared with controls without T2D, within the subset of study participants developing incident PAD during follow-up. Traditional ASCVD risk factors, HbA1c, and high age were important risk factors associated with incident all-cause and ASCVD mortality in persons with T2D and concomitant PAD.

### Electronic supplementary material

Below is the link to the electronic supplementary material.

Supplementary Material 1

## Data Availability

No datasets were generated or analysed during the current study.

## Abbreviations

ASCVD: Atherosclerotic cardiovascular disease
DM: Diabetes mellitus
GBM: Gradient-Boosting Model
ICD codes: International Classification of Diagnoses codes
LISA: The Longitudinal Integration Database for Health Insurance
MICE: Multiple imputations by chained equations
NDR: The Swedish National Diabetes Register
NPR: The National Patient Register
PAD: Peripheral artery disease
PDR: The Prescribed Drug Register
SMD: Standardized mean difference
T2D: Type 2 diabetes mellitus
TPR: The Total Population Register
