# Supplementary material for: Risk factors for and risk of all-cause and atherosclerotic cardiovascular disease mortality in people with type 2 diabetes and peripheral artery disease: an observational, register-based cohort study
Source: Cardiovasc Diabetol. 2024 Apr 15;23:127. doi: 10.1186/s12933-024-02226-x (PMC11020313; doi:10.1186/s12933-024-02226-x)
Supplement: Supplementary file 1 — Supplementary Material 1 [file 12933_2024_2226_MOESM1_ESM.docx]

**Risk factors for and risk of all-cause and atherosclerotic cardiovascular disease mortality in people with type 2 diabetes and peripheral artery disease: an observational, register-based cohort study**

Tarik Avdic, Hanne K. Carlsen, Aidin Rawshani, Soffia Gudbjörnsdottir, Zacharias Mandalenakis, Björn Eliasson

**Data Supplement**

**SUPPLEMENTARY MATERIAL 1**

**Table S1. ICD-10 Codes used to define baseline conditions and outcomes 3**

**Table S2. Variables assessed in the imputation model 4**

**Table S3. Supplementary Table 4. Predictors for all-cause mortality in persons with type**

**2 diabetes and peripheral artery disease 5**

**Table S4. Supplementary Table 5. Predictors for atherosclerotic cardiovascular disease**

**mortality after in persons with type 2 diabetes and peripheral artery disease 6**

**Supplementary Figure S1. Unadjusted survival curves of all-cause mortality in people with**

**type 2 diabetes versus controls 7**

**Supplementary Figure S2. Unadjusted survival curves of all-cause mortality in women with**

**type 2 diabetes versus controls 8**

**Supplementary Figure S3. Unadjusted survival curves of all-cause mortality in men with**

**type 2 diabetes versus controls 9**

**Supplementary Figure S4. Unadjusted survival curves of atherosclerotic cardiovascular disease mortality in people with type 2 diabetes versus controls 10**

**Supplementary Figure S5. Unadjusted survival curves of atherosclerotic cardiovascular disease mortality in women with type 2 diabetes versus controls 11**

**Supplementary Figure S6. Unadjusted survival curves of atherosclerotic cardiovascular disease mortality in men with type 2 diabetes versus controls 12**

**Supplementary Table I:** ICD Codes, 10^th^ revision of International Classification Disease codes (ICD-codes)

| **Diagnosis** | **ICD-10 Codes** |
| --- | --- |
| Acute myocardial infarction | I21 |
| Ischemic heart disease | I20-I25 |
| Stroke (all types) | I61-I64 |
| Atrial fibrillation | I48 |
| Heart failure | I50 |
| Diabetic complication. Hyperglycemia | E11.0, E11.1, E12.0, E12.1, E13.0, E13.1, E14.0, E14.1, |
| Atherosclerotic Cardiovascular Disease | I20-I25, I61-I64 |
| Psychiatric disease | F20-F29, F30-F30 |
| Cancer | C00-C97 |
| Hemorrhagic stroke | I61-I62 |
| Ischemic stroke | I63 |
| Non-specific stroke | I64 |
| Acute kidney failure and Chronic kidney disease | N17-N19 |
| Peripheral artery disease | I702, I702A, I702C, I702D, I702E, I702X, I739, I739B, I739C, I739X, I739W, I700 |
| Percutaneous coronary intervention | FNG00, FNG02, FNG05, FNG06, FNG10, FNG30, FNG96 |
| Coronary artery bypass graft | FNA00, FNA10, FNA20, FNA96, FNB00, FNB20, FNB96, FNC10, FNC20, FNC30, FNC40, FNC50, FNC60, FNC96, FND10, FND20, FND96, FNE00, FNE10, FNE20, FNE96, FNF00, FNF10, FNF20, FNF30, FNF96 |
| Valvular disease | I05-I09, I34-I37, Q22-Q23 |
| Amputation | NHQ09, NHQ11, NGQ09, NGQ11, NGQ19, NGQ99, NFQ09, NFQ19, NFQ99, NEQ19, NEQ99 |

ICD indicates International Classification of Disease. Atherosclerotic Cardiovascular Disease includes ischemic heart disease, stroke, and acute myocardial infarction

**Supplementary Table 2**: Variables assessed in the imputation model

| Variables assessed in the imputation model |
| --- |
| Age, sex, age at onset of diabetes, clinicians diagnosis type, treatment of diabetes, systolic blood pressure, diastolic blood pressure, body weight, body length, glycated hemoglobin (HbA1c), total cholesterol, triglycerides, HDL-cholesterol, LDL-cholesterol, albuminuria, s-creatinine, smoking status, physical activity, county, body mass index, marital status, education, ethnicity, income, eGFR, history of ASCVD, ischemic heart disease, myocardial infarction, peripheral artery disease, stroke, atrial fibrillation, heart failure, valvular disease, acute kidney failure and chronic kidney disease, psychiatric disease, cancer, treatment with anti-hypertensive medication, statins and antiplatelet therapy (aspirin included) and anticoagulant medication |

**Supplementary Table 3.** Predictors for all-cause mortality in persons with type 2 diabetes and peripheral artery disease

| **Variable (Risk Factor)** | **Hazard Ratio (95% CI)*** |
| --- | --- |
| Age, y | \| 1.08 \| (1.07- \| 1.08) \| † \|  \| \| --- \| --- \| --- \| --- \| --- \| |
| Sex |  |
| Female | \| 0.8 \| (0.74–0.87) \| † \|  \| \| --- \| --- \| --- \| --- \| |
| T2D duration, y | \| 1 \| (1- \| 1.01) \| \| --- \| --- \| --- \| |
| Antihyperglycemic therapy |  |
| Oral agents | \| 1.08 \| (0.99- \| 1.18) \| \| --- \| --- \| --- \| |
| Insulin | \| 1.26 \| ( \| 1.1-1.43) \| † \|  \| \| --- \| --- \| --- \| --- \| --- \| |
| Insulin and oral agents | \| 1.22 ( \| 1.08- \| 1.38) \| † \|  \| \| --- \| --- \| --- \| --- \| --- \| |
| HbA1c, mmol/mol | \| 1.01 \| (1- \| 1.01) \| \| --- \| --- \| --- \| |
| Blood pressure, mm Hg |  |
| Systolic | \| 1 \| (1- \| 1) \| \| --- \| --- \| --- \| |
| Diastolic | \| 1 \| (1- \| 1.01) \| \| --- \| --- \| --- \| |
| LDL-cholesterol, mmol/L | \| 1 \| .08 (0.97- \|  \| 1.2) \| \| --- \| --- \| --- \| --- \| |
| HDL-cholesterol, mmol/L | \| 1.06 \| (0.94- \| 1.2) \| \| --- \| --- \| --- \| |
| Triglycerides, mmol/L | \| 1.03 \| (0.98- \| 1.09) \| \| --- \| --- \| --- \| |
| BMI | \| 1 \| (0.99- \| 1.01) \| \| --- \| --- \| --- \| |
| eGFR, mL·min^−^1/1.73 m^−2^ | 1 (1-1) |
| Smoker, n (%) | \| 1.3 \| 2 (1.21 \| -1.44) \| † \|  \| \| --- \| --- \| --- \| --- \| --- \| |
| Medication, n (%) |  |
| Antihypertensive | \| 1.29 \| (1.17- \| 1.42) \| † \|  \| \| --- \| --- \| --- \| --- \| --- \| |
| Antiplatelet | \| 0.94 \| (0.86 \| -1.02) \| \| --- \| --- \| --- \| |
| Statins | \| 0.92 \| (0.85- \| 1) \| \| --- \| --- \| --- \| |
| Anticoagulant medication | \| 1.19 \| (1.04 \| -1.35) \| † \|  \| \| --- \| --- \| --- \| --- \| --- \| |
| Marital status, n (%) |  |
| Single | \| 1.15 (1. \| 05- \| 1.26) \| † \|  \| \| --- \| --- \| --- \| --- \| --- \| |
| Divorced | \| 1.28 (1.16-1.41) \| † \|  \| \| --- \| --- \| --- \| |
| Widowed | \| 1.24 \| (1.1- \| 1.39) \| † \|  \| \| --- \| --- \| --- \| --- \| --- \| |
| Country of birth, n (%) |  |
| Europe | \| 1 \| (0.87 \| -1.14) \| \| --- \| --- \| --- \| |
| Rest of the world | \| 0.78 \| (0.67- \| 0.89) \| † \|  \| \| --- \| --- \| --- \| --- \| --- \| |
| Income quartile n (%) |  |
| 2^nd^ quartile | \| 0.87 \| (0.8- \| 0.95) \| † \|  \| \| --- \| --- \| --- \| --- \| --- \| |
| 3^rd^ quartile | \| 0.82 \| (0.74- \| 0.91) \| † \|  \| \| --- \| --- \| --- \| --- \| --- \| |
| 4^th^ quartile | \| 0.72 \| (0.64- \| 0.82) \| † \|  \| \| --- \| --- \| --- \| --- \| --- \| |
| History of comorbidities, n (%) |  |
| Ischemic heart disease | \| 0.93 (0.85-1.02) \| \| --- \| |
| Stroke | 1.12 (0.98-1.28) |
| Cardiovascular disease | \| 1.24 \| (1.1- \| 1.38) \| † \|  \| \| --- \| --- \| --- \| --- \| --- \| |
| Atrial fibrillation | \| 1.22 \| (1.09- \| 1.37) \| † \|  \| \| --- \| --- \| --- \| --- \| --- \| |
| Heart failure | \| 1.5 \| (1.34- \| 1.67) \| † \|  \| \| --- \| --- \| --- \| --- \| --- \| |
| Valvular disease | \| 1.25 \| (1.06- \| 1.47) \| † \|  \| \| --- \| --- \| --- \| --- \| --- \| |
| Psychiatric disease | \| 1.48 \| (1.23-1.79) \| † \|  \| \| --- \| --- \| --- \| --- \| |
| Acute kidney failure and Chronic kidney disease | \| 1.55 \| (1.27- \| 1.9) \| † \|  \| \| --- \| --- \| --- \| --- \| --- \| |
| Cancer | \| 1.21 ( \| 1.1- \| 1.33) \| † \|  \| \| --- \| --- \| --- \| --- \| --- \| |

**Legend:** *Based on a multivariate Cox regression analysis for all persons with T2D and PAD excluding those with pre-study PAD events. Adjustment was made for all variables under column “T2D with PAD” shown in Table 1. †Significant difference versus controls (*P*<0.05). BMI indicates body mass index; eGFR, estimated glomerular filtration rate; HbA1c, glycated hemoglobin; HDL-C, high-density lipoprotein cholesterol; LDL-C, low-density lipoprotein cholesterol; and T2D, type 2 diabetes mellitus

**Supplementary Table 4.** Predictors for atherosclerotic cardiovascular disease mortality in persons with type 2 diabetes and peripheral artery disease

| **Variable (Risk Factor)** | **Hazard Ratio (95% CI)*** |
| --- | --- |
| Age, y | \| 1.08 \| (1.07 \| -1.09) \| † \|  \| \| --- \| --- \| --- \| --- \| --- \| |
| Sex |  |
| Female | \| 0.79 \| (0.73 \| -0.87) \| † \|  \| \| --- \| --- \| --- \| --- \| --- \| |
| T2D duration, y | \| 1 \| (1 \| -1.01) \| \| --- \| --- \| --- \| |
| Antihyperglycemic therapy |  |
| Oral agents | \| 1.08 \| (0.98 \| -1.18) \| \| --- \| --- \| --- \| |
| Insulin | \| 1.25 \| (1.09 \| -1.43) \| † \|  \| \| --- \| --- \| --- \| --- \| --- \| |
| Insulin and oral agents | \| 1.21 \| (1.06- \| 1.37) \| † \|  \| \| --- \| --- \| --- \| --- \| --- \| |
| HbA1c, mmol/mol | \| 1.01 \| (1- \| 1.01) \| \| --- \| --- \| --- \| |
| Blood pressure, mm Hg |  |
| Systolic | \| 1 \| (1 \| -1) \| \| --- \| --- \| --- \| |
| Diastolic | \| 1 \| (1 \| -1.01) \| \| --- \| --- \| --- \| |
| LDL-cholesterol, mmol/L | \| 1.07 \| (0.96 \| -1.2) \| \| --- \| --- \| --- \| |
| HDL-cholesterol, mmol/L | \| 1.05 \| (0.93- \| 1.19) \| \| --- \| --- \| --- \| |
| Triglycerides, mmol/L | \| 1.03 \| (0.97- \| 1.08) \| \| --- \| --- \| --- \| |
| BMI | \| 1 \| (1- \| 1.01) \| \| --- \| --- \| --- \| |
| eGFR, mL·min^−^1/1.73 m^−2^ | \| 1 ( \| 1- \| 1) \| \| --- \| --- \| --- \| |
| Smoker, n (%) | \| 1.31 \| (1.19- \| 1.44) \| † \|  \| \| --- \| --- \| --- \| --- \| --- \| |
| Medication, n (%) |  |
| Antihypertensive | \| 1.28 \| (1.16- \| 1.41) \| † \|  \| \| --- \| --- \| --- \| --- \| --- \| |
| Antiplatelet | \| 0.93 \| (0.85- \| 1.01) \|  \|  \| \| --- \| --- \| --- \| --- \| --- \| |
| Statins | \| 0.93 ( \| 0.85- \| 1.01) \|  \|  \| \| --- \| --- \| --- \| --- \| --- \| |
| Anticoagulant medication | \| 1.2 \| (1.04- \| 1.38) \| † \|  \| \| --- \| --- \| --- \| --- \| --- \| |
| Marital status, n (%) |  |
| Single | \| 1.15 \| (1.04- \| 1.26) \| † \|  \| \| --- \| --- \| --- \| --- \| --- \| |
| Divorced | \| 1.27 \| (1.15- \| 1.41) \| † \|  \| \| --- \| --- \| --- \| --- \| --- \| |
| Widowed | \| 1.23 \| (1.08- \| 1.4) \| † \|  \| \| --- \| --- \| --- \| --- \| --- \| |
| Country of birth, n (%) |  |
| Europe | \| 0.97 \| (0.84 \| -1.12) \| \| --- \| --- \| --- \| |
| Rest of the world | \| 0.75 \| (0.65- \| 0.87) \| † \|  \| \| --- \| --- \| --- \| --- \| --- \| |
| Income quartile n (%) |  |
| 2^nd^ quartile | \| 0.87 \| (0.79- \| 0.95) \| † \|  \| \| --- \| --- \| --- \| --- \| --- \| |
| 3^rd^ quartile | \| 0.82 ( \| 0.73 \| -0.91) \| † \|  \| \| --- \| --- \| --- \| --- \| --- \| |
| 4^th^ quartile | \| 0.75 \| (0.65- \| 0.87) \| † \|  \| \| --- \| --- \| --- \| --- \| --- \| |
| History of comorbidities, n (%) |  |
| Ischemic heart disease | \| 0.92 \| (0.83- \| 1.01) \| \| --- \| --- \| --- \| |
| Stroke | \| 1.11 \| (0.97 \| -1.29) \| \| --- \| --- \| --- \| |
| Cardiovascular disease | \| 1.24 \| (1.1 \| -1.4) \| † \|  \| \| --- \| --- \| --- \| --- \| --- \| |
| Atrial fibrillation | \| 1.23 \| (1.09 \| -1.39) \| † \|  \| \| --- \| --- \| --- \| --- \| --- \| |
| Heart failure | \| 1.5 \| (1.33- \| 1.69) \| † \|  \| \| --- \| --- \| --- \| --- \| --- \| |
| Valvular disease | \| 1.28 \| (1.08- \| 1.52) \| † \|  \| \| --- \| --- \| --- \| --- \| --- \| |
| Psychiatric disease | \| 1.51 \| (1.24 \| -1.83) \| † \|  \| \| --- \| --- \| --- \| --- \| --- \| |
| Acute kidney failure and Chronic kidney disease | \| 1.51 \| (1.22- \| 1.87) \| † \|  \| \| --- \| --- \| --- \| --- \| --- \| |
| Cancer | \| 1.22 \| (1.11-1.35) \| † \|  \| \| --- \| --- \| --- \| --- \| |

**Legend:** *Based on a multivariate Cox regression analysis for all persons with T2D and PAD excluding those with pre-study PAD events. Adjustment was made for all variables under column “T2D with PAD” shown in Table 1.

†Significant difference versus controls (*P*<0.05). BMI indicates body mass index; eGFR, estimated glomerular filtration rate; HbA1c, glycated hemoglobin; HDL-C, high-density lipoprotein cholesterol; LDL-C, low-density lipoprotein cholesterol; and T2D, type 2 diabetes mellitus

**
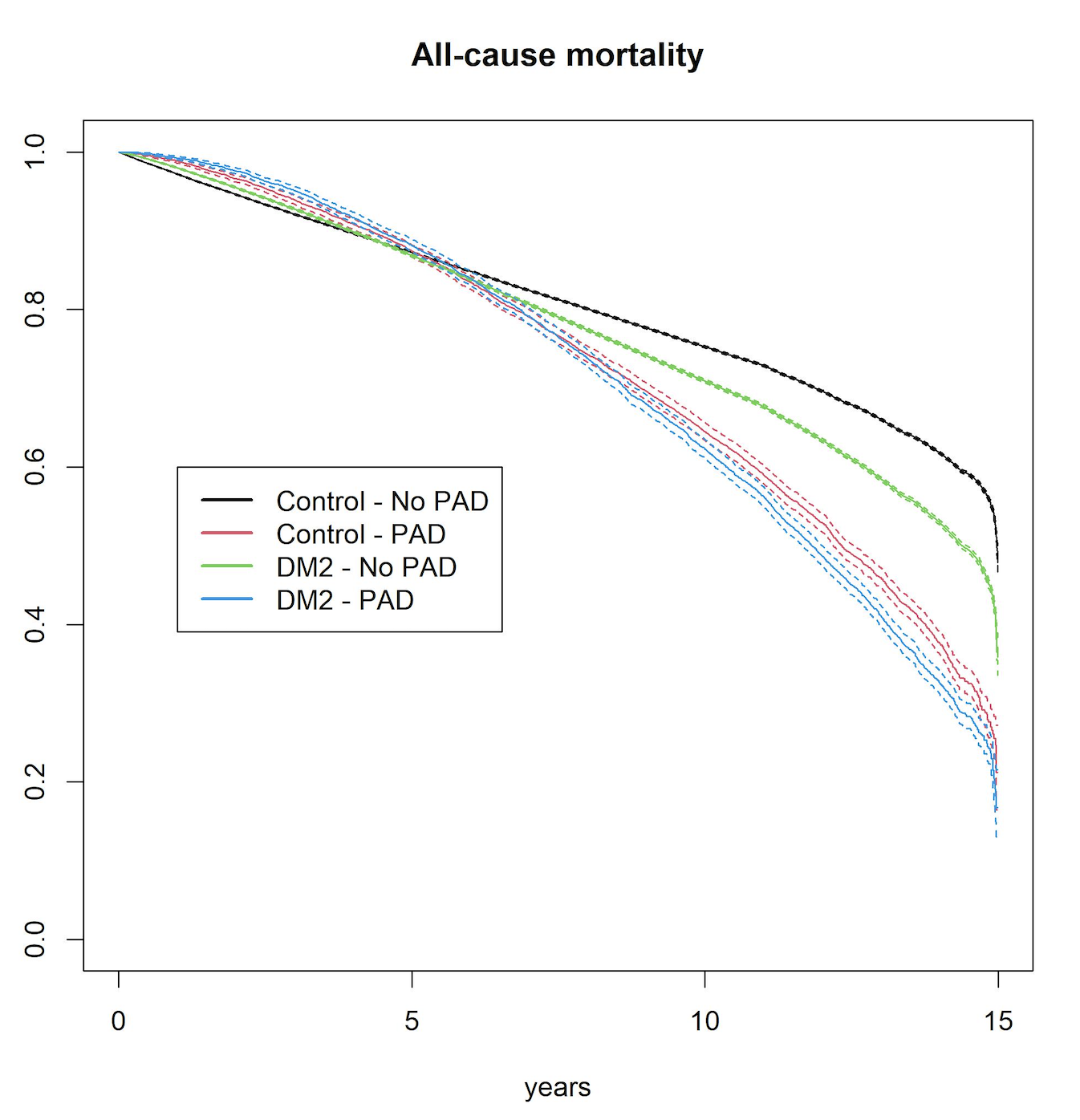
Supplementary Figure 1.** Unadjusted survival curves of all-cause mortality in people with type 2 diabetes versus controls

**Legend:** Kaplan-Meier curves presenting the unadjusted survival curves of all-cause mortality in people with type 2 diabetes versus controls, stratified whether onset of incident peripheral artery disease occurred or not**.** PAD indicates peripheral artery disease; and DM 2 indicates type 2 diabetes

**Supplementary Figure 2.** Unadjusted survival curves of all-cause mortality in women with type 2 diabetes versus controls

**
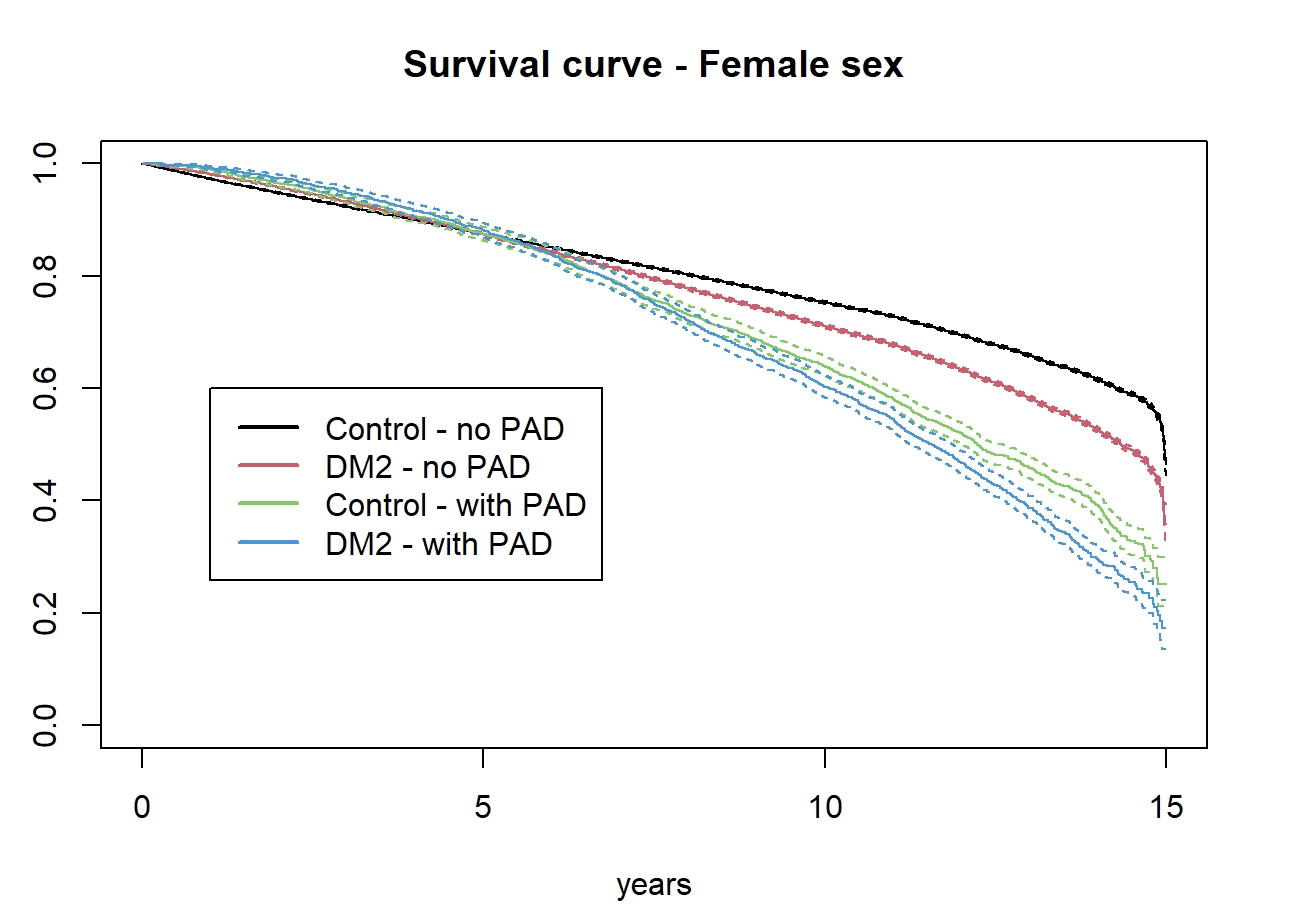
**

**Legend:** Kaplan-Meier curves presenting the unadjusted survival curves of all-cause mortality in women with type 2 diabetes versus controls, stratified whether onset of incident peripheral artery disease occurred or not**.** PAD indicates peripheral artery disease; and DM 2 indicates type 2 diabetes

**Supplementary Figure 3.** Unadjusted survival curves of all-cause mortality in men with type 2 diabetes versus controls

**
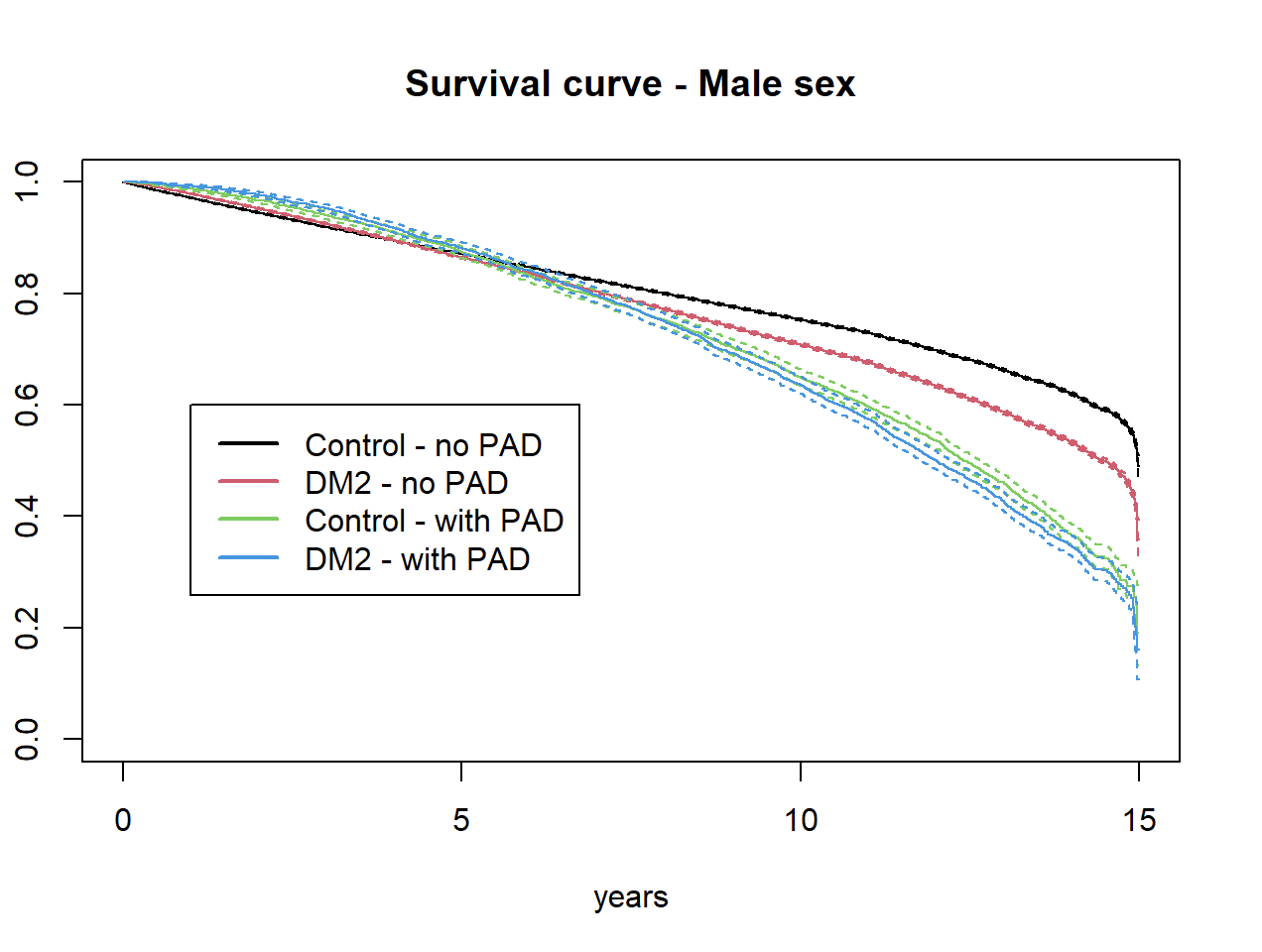
**

**Legend:** Kaplan-Meier curves presenting the unadjusted survival curves of all-cause mortality in men with type 2 diabetes versus controls, stratified whether onset of incident peripheral artery disease occurred or not**.** PAD indicates peripheral artery disease; and DM 2 indicates type 2 diabetes


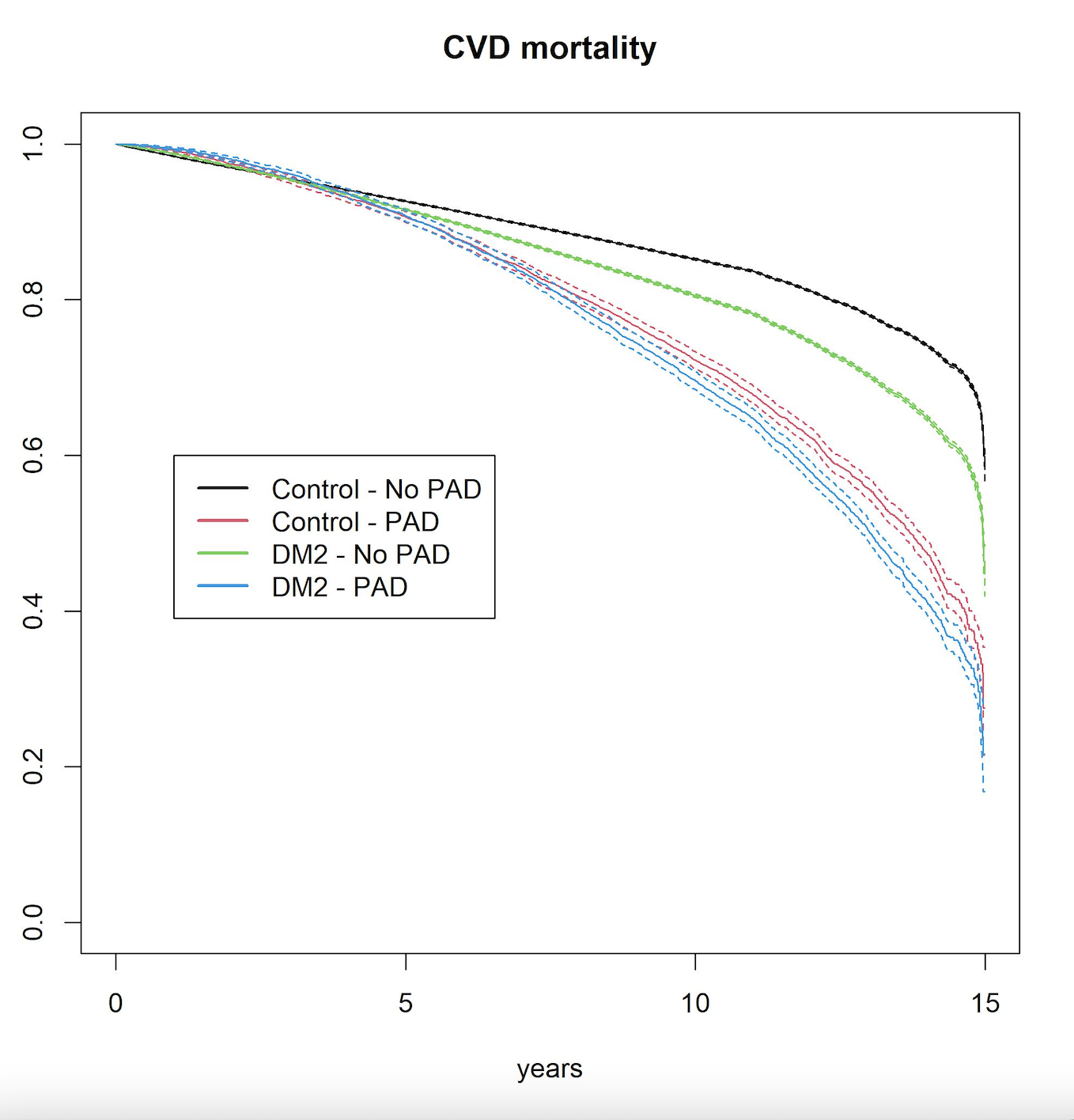
**Supplementary Figure 4.** Unadjusted survival curves of atherosclerotic cardiovascular disease mortality in people with type 2 diabetes versus controls

**Legend:** Kaplan-Meier curves presenting the unadjusted survival curves of atherosclerotic cardiovascular disease mortality in people with type 2 diabetes versus controls, stratified by onset of incident peripheral artery disease PAD indicates peripheral artery disease; CVD indicates cardiovascular and DM 2 indicates type 2 diabetes

**Supplementary Figure 5.** Unadjusted survival curves of atherosclerotic cardiovascular disease mortality in women with type 2 diabetes versus controls


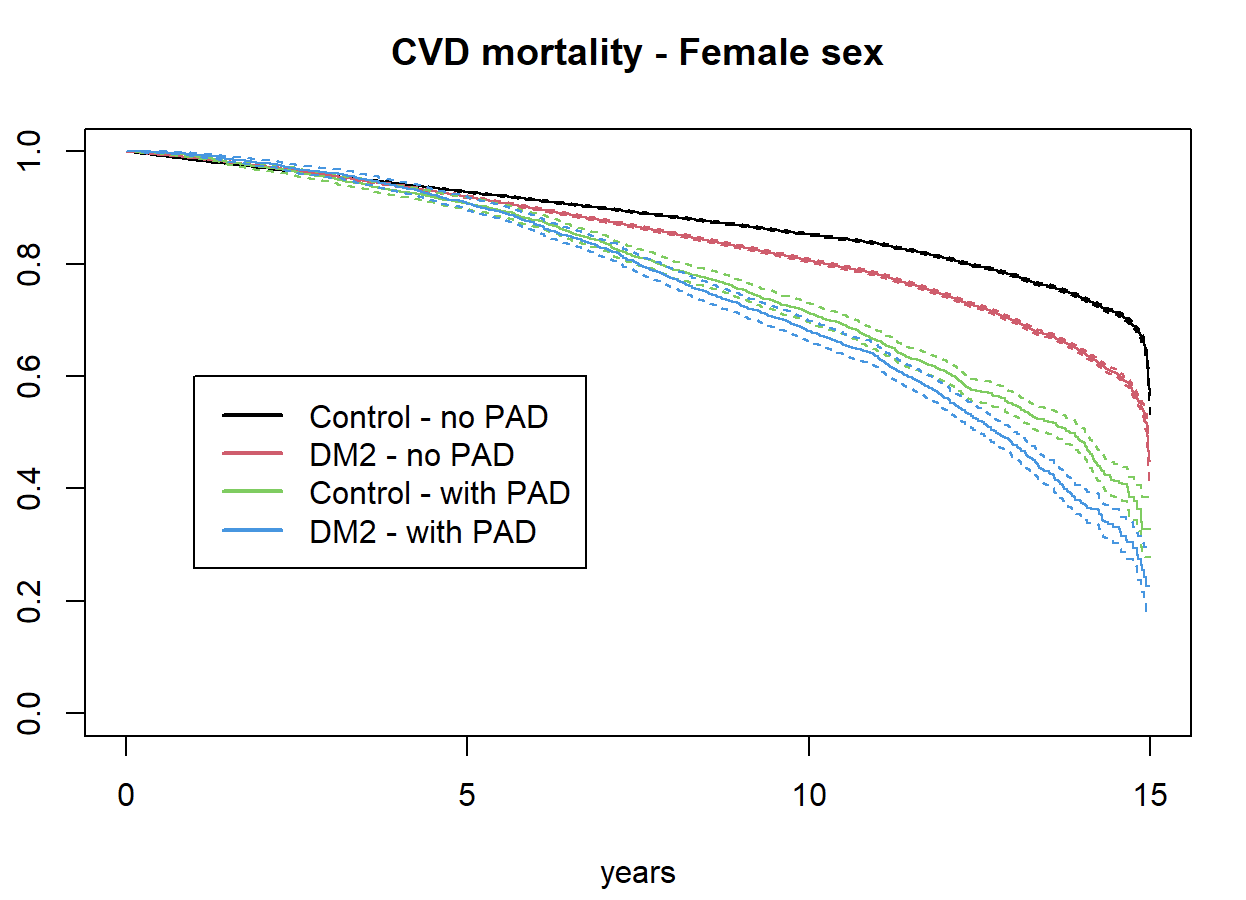


**Legend:** Kaplan-Meier curves presenting the unadjusted survival curves of atherosclerotic cardiovascular disease mortality in women with type 2 diabetes versus controls, stratified by onset of incident peripheral artery disease PAD indicates peripheral artery disease; CVD indicates cardiovascular and DM 2 indicates type 2 diabetes

**Supplementary Figure 6.** Unadjusted survival curves of atherosclerotic cardiovascular disease mortality in men with type 2 diabetes versus controls


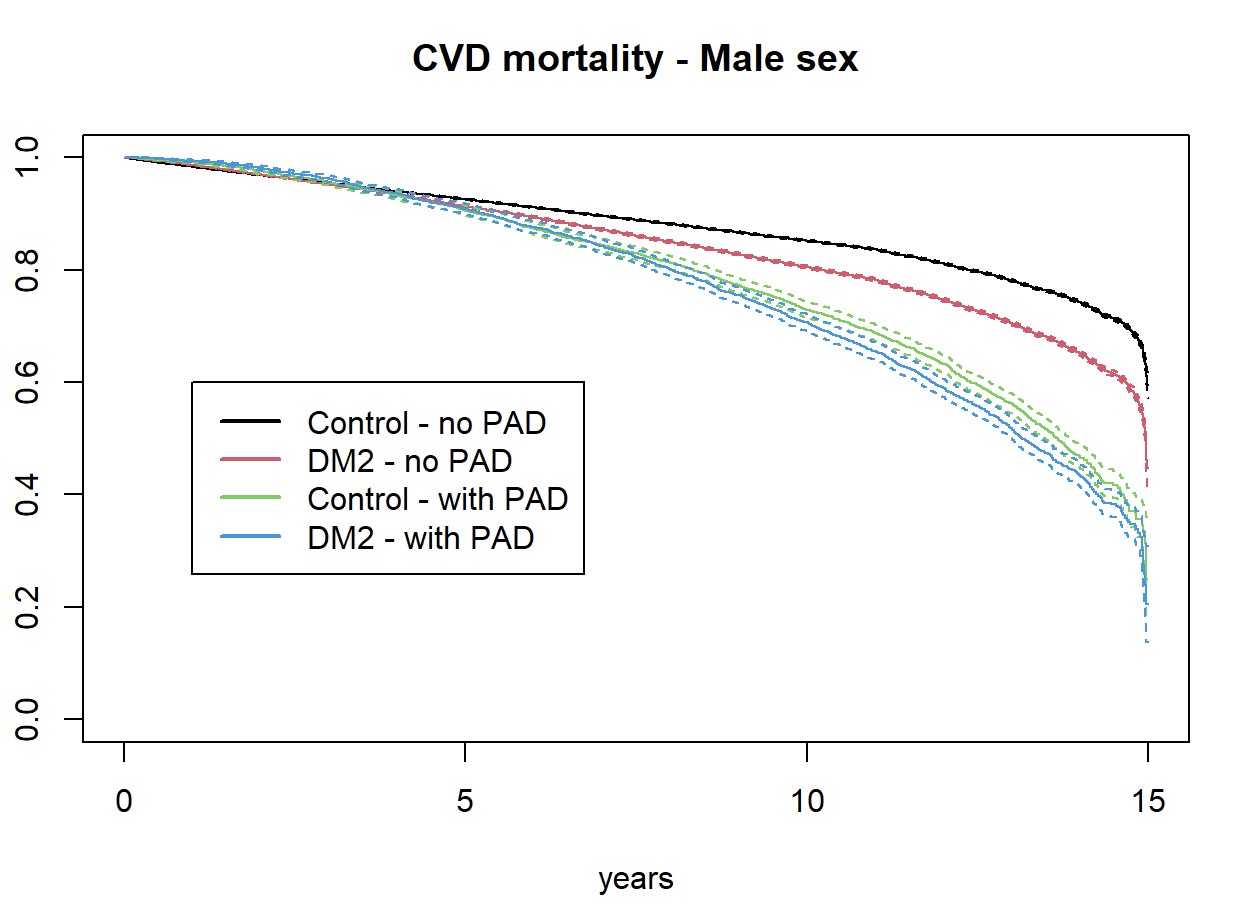


**Legend:** Kaplan-Meier curves presenting the unadjusted survival curves of atherosclerotic cardiovascular disease mortality in men with type 2 diabetes versus controls, stratified by onset of incident peripheral artery disease PAD indicates peripheral artery disease; CVD indicates cardiovascular and DM 2 indicates type 2 diabetes
